# Gaps and strategies in developing health research capacity: experience from the Nigeria Implementation Science Alliance

**DOI:** 10.1186/s12961-018-0289-x

**Published:** 2018-02-12

**Authors:** Echezona E. Ezeanolue, William Nii Ayitey Menson, Dina Patel, Gregory Aarons, Ayodotun Olutola, Michael Obiefune, Patrick Dakum, Prosper Okonkwo, Bola Gobir, Timothy Akinmurele, Anthea Nwandu, Hadiza Khamofu, Bolanle Oyeledun, Muyiwa Aina, Andy Eyo, Obinna Oleribe, Ikoedem Ibanga, John Oko, Chukwuma Anyaike, John Idoko, Muktar H. Aliyu, Rachel Sturke, Nigeria Implementation Science Alliance

**Affiliations:** 1 School of Community Health Sciences, University of Nevada Las Vegas, Las Vegas, NV United States of America; 2 Healthy Sunrise Foundation, Las Vegas, NV United States of America; 3 University of California San Diego, San Diego, CA United States of America; 4 Centre for Clinical Care and Clinical Research, Abuja, Nigeria; 5 Institute of Human Virology, University of Maryland, Baltimore, MD United States of America; 6 Institute of Human Virology, Abuja, Nigeria; 7 AIDS Prevention Initiative, Abuja, Nigeria; 8 Maryland Global Initiatives Corporation, Baltimore, MD United States of America; 9 Enhanced Health Access Initiatives, Abuja, Nigeria; 10 Family Health International, Abuja, Nigeria; 11 Center for Integrated Health Programs, Abuja, Nigeria; 12 Solina Health, Abuja, Nigeria; 13 Excellence Community Education Welfare Scheme, Abuja, Nigeria; 14 Excellence and Friends Management Consult, Abuja, Nigeria; 15 ProHealth International, Abuja, Nigeria; 16 Catholic Caritas Foundation Nigeria, Abuja, Nigeria; 17 Federal Ministry of Health, Abuja, Nigeria; 18 National Agency for Control of AIDS, Abuja, Nigeria; 19 Vanderbilt Institute for Global Health, Vanderbilt University Medical Center, Nashville, TN United States of America; 20 Fogarty International Center, National Institutes of Health, Bethesda, MD United States of America

**Keywords:** Research capacity, Implementation science, Collaborative research, Health

## Abstract

**Background:**

Despite being disproportionately burdened by preventable diseases than more advanced countries, low- and middle-income countries (LMICs) continue to trail behind other parts of the world in the number, quality and impact of scholarly activities by their health researchers. Our strategy at the Nigerian Implementation Science Alliance (NISA) is to utilise innovative platforms that catalyse collaboration, enhance communication between different stakeholders, and promote the uptake of evidence-based interventions in improving healthcare delivery. This article reports on findings from a structured group exercise conducted at the 2016 NISA Conference to identify (1) gaps in developing research capacity and (2) potential strategies to address these gaps.

**Methods:**

A 1-hour structured group exercise was conducted with 15 groups of 2–9 individuals (*n* = 94) to brainstorm gaps for implementation, strategies to address gaps and to rank their top 3 in each category. Qualitative thematic analysis was used. First, duplicate responses were merged and analyses identified emerging themes. Each of the gaps and strategies identified were categorised as falling into the purview of policy-makers, researchers, implementing partners or multiple groups.

**Results:**

Participating stakeholders identified 98 gaps and 91 strategies related to increasing research capacity in Nigeria. A total of 45 gaps and an equal number of strategies were ranked; 39 gaps and 43 strategies were then analysed, from which 8 recurring themes emerged for gaps (lack of sufficient funding, poor research focus in education, inadequate mentorship and training, inadequate research infrastructure, lack of collaboration between researchers, research-policy dissonance, lack of motivation for research, lack of leadership buy-in for research) and 7 themes emerged for strategies (increased funding for research, improved research education, improved mentorship and training, improved infrastructure for research, increased collaboration between academic/research institutions, greater engagement between researchers and policy-makers, greater leadership buy-in for research).

**Conclusions:**

The gaps and strategies identified in this study represent pathways judged to be important in increasing research and implementation science capacity in Nigeria. The inclusion of perspectives and involvement of stakeholders who play different roles in policy, research and implementation activities makes these findings comprehensive, relevant and actionable, not only in Nigeria but in other similar LMICs.

**Electronic supplementary material:**

The online version of this article (10.1186/s12961-018-0289-x) contains supplementary material, which is available to authorized users.

## Background

In spite of the high burden of disease in low- and middle-income countries (LMICs) compared to more prosperous parts of the world [1], health researchers in LMICs continue to trail behind their counterparts in more developed settings with regards to the number, quality and impact of their scholarly activities [2]. Nigeria is Africa’s most populous country and, while relatively better-resourced in academic infrastructure and scientific research productivity than many other countries in sub-Saharan Africa, its health status indices remain disappointingly poor [3]. Prevailing public health challenges, such as high levels of maternal and child mortality, infectious disease outbreaks, a plethora of endemic infectious conditions, substantial burden of mother-to-child HIV transmission, and a rising incidence of non-communicable diseases, constitute a major hindrance to the attainment of national health targets. To compound matters, weak linkages between research and policy [4] contribute to delays in the timely and efficient adoption and implementation of evidence-based practices and methodologies, which in turn limit the development of a resilient and responsive national health system. A deliberate, robust and sustained approach to building local scientific research capacity and strengthening evidence-based policy-making and practice is critical to overcoming these challenges.

Implementation science is an emerging field of study that seeks to bridge the research-to-practice gap via integration of research findings and other evidence-based practices into routine care and services [5]. There are numerous implementation science approaches that can be strategically employed to sustainably increase scientific research capacity and bridge gaps in incorporating research evidence into decision-making processes. One strategy is to utilise innovative platforms that catalyse collaboration and enhance communication between researchers, policy-makers and health programme implementers [6]. The Nigeria Implementation Science Alliance (NISA) was established in 2015 as a robust partnership of 20 local organisations comprising researchers, programme implementers and policy-makers. The aim of NISA is to provide a forum to facilitate discussion and actions related to cross-cutting implementation science issues and to identify research-to-policy gaps and approaches that are feasible, culturally appropriate and relevant to the Nigeria environment as well as to promote actionable strategies to improve public health [7]. This is modelled after the NIH-PEPFAR PMTCT Implementation Science Alliance, which uses a similar strategy to prevent mother-to-child transmission of HIV [6].

The second meeting of the NISA was held in Abuja, Nigeria, in September 2016. The meeting focused on identifying challenges in conducting health research and in incorporating evidence from health research into policy and practice in Nigeria. Attendees also proffered solutions to addressing challenges and bridging existing gaps in the research-to-policy-to-practice continuum. This paper documents findings arising from the conference, and provides important insights into the challenges faced by Nigerian researchers, academia, programme implementers and policy-makers in scaling locally led health research and their recommendations as to how to achieve a reliable health system that supports evidence-based policy-making and practice.

## Methods

### Process and participants

A 1-hour structured modified nominal group process (NGP) exercise [8] was conducted to identify and prioritise (1) gaps in developing research capacity and (2) potential strategies to address the gaps identified. Ninety-four individuals participated in this NGP in 15 groups of 2 to 9 individuals (average group size = 6 individuals). Group members included representatives from PEPFAR implementing partners, academia, researchers, clinicians and policy-makers. Among the 94 participants in this NGP exercise, 40 were programme implementing partners, 5 were policy-makers and 13 were researchers. Some participants were identified as belonging to multiple categories, either as both implementing partner and researcher (*n* = 24), researcher and policy-maker (*n* = 5), implementing partner and policy-maker (*n* = 1), or implementing partner, researcher and policy-maker (*n* = 6) (Table 1).

**Table 1:** Characteristics of participants

Group	Number
Policy-makers	5
Implementing partners (IP)	40
Research institutions	13
Policy-maker and IP	1
Policy-maker and researcher	5
IP and researcher	24
Policy-maker and researcher and IP	6

The NGP was conducted in two 20-minute phases designed to maximise participant focus and engagement and had three components. During the first 20 minutes, each group member identified gaps in developing research capacity in Nigeria and then the groups discussed and elaborated on these issues. The second 20-minute phase focused on identifying strategies to address the gaps identified in the previous session. Each of these sessions included three distinct activities, namely (1) generating ideas (i.e. gaps or strategies), (2) listing ideas (i.e. gaps or strategies, and (3) ranking the gaps and strategies identified in the first two steps. The first activity, ‘brainstorming’, utilised as part of this NGP, is an effective and low-cost method of identifying plausible implementation gaps and potential solutions to these gaps [9]. After generating a list of items, groups were asked to rank the top three by order of importance in each category (gaps and strategies). Groups independently identified between 2 and 9 gaps and between 3 and 9 strategies. Thus, the exercise yielded a total of 98 gaps and 45 ranked items (3 gaps × 15 groups) and a similar number of strategies (91 total, 45 ranked strategies) to address the gaps identified. During the last part of the exercise, groups selected a representative to share their group’s top priority in each category with the other groups. After the exercise, the groups’ priorities were collected, collated and later transcribed for data analysis and interpretation.

### Data analysis

**Fig. 1:**
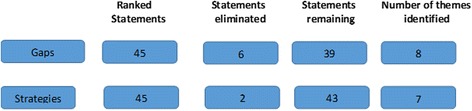
Statement generation and consolidation. A description of the process of ranking statements and organising them by thematic areas

We analysed de-identified data and reported aggregated results. On these grounds, this research was approved as exempt. In the first phase of data analysis, all responses were transcribed and entered into an Excel database and sorted to identify duplicate entries. Following this, three research team members with expertise in policy, research and implementation reviewed and eliminated statements that were deemed to be invalid or duplicate. These responses were then combined based on recurring themes by five public health practitioners with expertise in research and policy [10]. For each gap that could be explained by more than two themes, the two themes that most strongly explained it were maintained. In the next phase, five other public health practitioners with relevant research experience categorised the identified gaps and strategies by the level (public policy, network of implementing partners and research institutions) at which the gap is occurring or where the strategy identified could be implemented. A sixth coder resolved any disagreements, making the ultimate decision about the appropriateness of the assigned levels and categories. Figure 1 depicts the process of generating, consolidating and categorising items from the structured exercise.

Gaps that were identified as applicable to the work of federal and state governments were categorised as public policy gaps. Those seen as soluble by the collaboration of different partners/organisations like NISA were classified as occurring at the level of network of implementing partners, and those that exist because of a problem in an institution or at an individual level were classified as institutional/researchers.

## Results

We identified eight overarching themes from the list of gaps and seven emerged from the list of strategies (Table 2). Specific gaps identified and grouped under particular themes were initially described using language reflecting the general role of the contributor. For example, under the theme ‘Lack of sufficient funding’, a policy-maker might document ‘Lack of access to funds for research’, whilst a researcher would write ‘Lack of awareness of research funding’. The specific points made by these stakeholders reflected their unique circumstances in appreciating these gaps. Specific nuances identified during the analysis of the strategies mentioned were grouped under broad categories, namely gaps and strategies.

**Table 2:** Overarching themes for gaps and strategies identified

Overarching themes	
Gaps	Strategies
1. Lack of commitment by different stakeholders to provide funding for research 2. Poor research focus in undergraduate and post-graduate education curriculum 3. Inadequate mentorship and training in research for early stage faculty 4. Inadequate infrastructure for research 5. Lack of collaboration/partnership within and between academic institutions, and between academic institutions and programme implementers 6. Research–policy dissonance 7. Lack of interest and motivation for research 8. Lack of leadership buy-in for research	1. Increased provision of funding for research by stakeholders, e.g. government 2. Increased research component in educational curriculum 3. Improved mentorship and training in research by senior faculty to junior faculty 4. Increased investment in infrastructure for research in higher education institutions 5. Creation of an enabling environment for collaboration within and between academic institutions and between academic institutions and programme implementers 6. Greater engagement between researchers and policy-makers 7. Greater commitment by institutional leadership for research

Of the gaps and strategies identified, stakeholders categorised five gaps as being solely public policy issues, four gaps as issues among the network of implementing partners and five as issues in research institutions (Table 3).

**Table 3:** Levels of gaps and strategies

Level	Number of gaps	Number of strategies
Public policy	5	6
Network of implementing partners (IPs)	4	4
Institutions/researchers	5	7
Public policy and IPs	1	1
Public policy and institutions	8	4
IPs and institutions	14	14
All three	2	7

Participants suggested increasing institutional budgetary allocations for research, increasing government financial support for research and the establishment of a research fund to bridge the gaps associated with the ‘lack of sufficient funding’ and ‘need for increased funding’ themes identified.

Poor research focus was also identified as a major gap in research capacity in LMICs. Participants provided examples of situations where lecturers were themselves not adequately trained in research and so were unable to impart the knowledge they did not have. Short courses for university lecturers as well as partnerships and mentorship by more experienced researchers in other institutions were identified as approaches in which this gap could be bridged. Since some graduate students might grow into researchers, earlier exposure to the rudiments of research was identified as a way of building future generations of competent LMIC researchers. Effective advocacy by the research community in educating the public and policy-makers on the importance of research was identified as a potentially effective tool for development of the critical infrastructure needed for the smooth conduct of research in LMIC institutions. Most contemporary research involves cross-cutting themes and therefore requires the collaboration of persons with diverse skillsets, programme implementers with sufficient on-ground infrastructure and policy-makers with control of much-needed resources. Finally, participants strongly suggested that the conduct of research be tied directly to promotion in academia. This is likely to motivate faculty to conduct research and foster their retention in research-focused careers.

Among the identified strategies, six were identified as being implementable at the public policy level, four at the level of implementing partners and seven at the research/institutional level. It is worthy of note that most of the gaps and strategies identified were applicable at more than one level, with two gaps and seven strategies categorised as applicable to all three levels (Table 3).

## Discussion

The modified NGP and thematic analysis results suggest that the sub-optimal research capacity in Nigeria is the result of several gaps in capacity in each of three domain areas, namely policy, research and implementation. The categorisation of these gaps and strategies is important in order to identify where these problems occur and how solutions to these can be devised as strategies. In addition, the results identified groups of stakeholders with the capacity and ability to implement some of the strategies identified and suggests specific roles in research capacity-building activities. In general, most of the factors identified are consistent with what is reported in previously published literature from other parts of the world, such as inadequate investment in research, a lack of motivation of individual researchers, inadequate training in research methods, and a lack of focused research responding to a society’s needs [11–13].

Twenty-five of the gaps identified were applicable to more than one group of stakeholders. This emphasises the need for the cultivation of closer relationships among different stakeholders, reaffirming one of the major motivations for the 2015 NISA conference, which sought to identify strategies for reducing mother-to-child transmission of HIV [7]. It also represents a direct opportunity for the cross-fertilisation of ideas and efforts in increasing research capacity. Many of the groups in this NGP recommended the formation of partnerships between institutions, programme implementers and policy-makers.

Cross-cutting gaps that were applicable to more than one stakeholder group included a lack of adequate infrastructure for research, a lack of training and mentorship, as well as a lack of strong leadership committed to research. These gaps convey a sense of shared responsibility among the different stakeholders and serve as a clarion call to academic institutions to make collaborative academic–public research an integral part of their activities.

It is noteworthy that, for some of the gaps identified and ranked, more than one strategy was identified, some of which were not ranked. This resulted in some ranked strategies that did not correspond to the gaps identified and ranked. which might be considered a limitation of the work conducted as it would be desirable to have a proposed solution or strategy to address each gap. However, this also points to the need for processes to develop strategies to address identified and emergent gaps.

A limited research focus and lack of training in research methods in the undergraduate and post-graduate curriculum of most Nigerian universities was cited as one of the major causes of poor research capacity. This often results in suboptimal research skills and therefore substandard quality of research designs and methods, which attenuates the potential impact of such research findings. To bridge this gap, participants suggested that educational institutions and policy-makers increase the research component of educational curricula in order for universities to promote productive high quality research careers for their students. Other methods of bridging this gap identified by participants were the organisation of research capacity-building workshops and in-service research training for instructors/faculty at tertiary education units.

In addition, the lack of adequate training and mentorship in research was identified as one of the major gaps to increasing research capacity. In the Nigerian context, the lack of a structured research mentoring system where experienced researchers mentor younger ones was seen as problematic in efforts to increase research capacity. This is consistent with findings from research in other parts of Africa and the global south [11, 14]. To bridge this gap, participants suggested leveraging platforms like NISA to build mentor–mentee relationships in order to transfer knowledge and skills to early career researchers. Other strategies, like the organisation of conferences and workshops as well as the establishment of an online interactive platform for information exchange, were described as viable strategies for building research capacity in academic and non-academic settings [15]. One group suggested that, for every research project, the most senior researcher have a mentee throughout, so that knowledge and skills can be transferred and a new generation of researchers nurtured.

A lack of research that addresses the needs of the community was another frequently occurring barrier identified by participants. Closely related to this identified gap was a policy–research dissonance where government policies were out of sync with research findings. Additionally, participants cited a non-conducive environment for dissemination of research findings as one of the reasons for this dissonance between policy and research. The strategy identified here was mainly for increased communication between researchers, programme implementers and policy-makers. However, the specifics of how to improve communication still needs to be further developed.

Poor infrastructure for research, which was cited as a key cause of poor environment for research, was closely associated with poor documentation of data from healthcare delivery and other related facilities due to outdated or an absence of health management information systems resulting in low-quality data. Increased investment in health management information systems, better documentation and training of service providers in these technologies were identified as potentially effective strategies to increase the research capacity of these facilities.

A lack of interest and motivation for research by individuals, institutions and governments was one of the most commonly identified gaps. To address this, it was recommended that academic institutions make the conduct of research by faculty a requirement for promotion. In addition, increased investment in infrastructure for the conduct of successful research was identified as having great potential to create a conducive environment that can spark an interest in research in staff of academic and other institutions. It was also suggested that institutions form strategic alliances and collaborations with governments and interested organisations to make grants available for the training and capacity-building of young researchers [16, 17]. Indeed, this is an intervention that has been successfully used to increase research capacity in Mali, as evidenced by the technical and financial assistance provided by the Special Programme for Research and Training in Tropical Diseases to establish the Malaria Research and Training Centre in Bamako, Mali [13].

To utilise the strategies recommended through this NGP, participants proposed greater engagement of researchers/academia and programme implementers with policy-makers, arguing that this will help investigators carry out more relevant research. In addition, it will enhance policy-makers’ appreciation of research and their commitment to support it and apply research findings to policy. Greater engagement at all levels is expected to lead to better buy-in of leaders at all levels, solving another gap identified, the lack of leadership buy-in for research. However, recent advances in leader development strategies could also help to address this gap [18]. This proposed strategy also finds support in the work of Brownson et al. [19], who recommended greater involvement in the process of policy-making, building effective teams and developing political champions. They also posit, like participants at this conference that “*scientists are obligated not only to discover new knowledge but also to ensure that discoveries are applied to improve health*”.

### Limitations

Our study has limitations. The NGP has a certain degree of inflexibility and requires some conformity. We tried to reduce these effects by synthesising the viewpoints of stakeholders with totally different roles and perspectives of research capacity. In addition, there was the potential for the loss of important detail because of nuanced interpretations of others’ viewpoints. Furthermore, we are aware that, in analysing issues such as policy, the authors’ background may skew the interpretation of results. To reduce the impact of this factor, we included authors of different backgrounds in order to balance out these all-important perspectives.

## Conclusion

The gaps and strategies identified involved the roles of different stakeholders with interests and commitment to improving public health through research, policy and practice. It is therefore our expectation that these findings would be used in creating a research policy document, with buy-in from different stakeholders. Taking into consideration countries’ unique circumstances, this will guide efforts to increase capacity for the conduct of all forms of research and particularly implementation science.

## Additional file

Additional file 1: Blank data collection form. (DOCX 22 kb)

## Abbreviations

LMIC: now- and middle-income country
NGP: nominal group process
NISA: Nigeria Implementation Science Alliance
