# Supplementary material for: Gaps and strategies in developing health research capacity: experience from the Nigeria Implementation Science Alliance
Source: Health Res Policy Syst. 2018 Feb 12;16:10. doi: 10.1186/s12961-018-0289-x (PMC5809995; doi:10.1186/s12961-018-0289-x)
Supplement: Supplementary file 1 — Blank data collection form. (DOCX 22 kb) [file 12961_2018_289_MOESM1_ESM.docx]

| **Make a list of all gaps in developing research capacity** | **Rank the top three** | **Make a list of all interventions to bridge the gap in developing research capacity** | **Rank the top three** |
| --- | --- | --- | --- |
|  |  |  |  |
|  |  |  |  |
|  |  |  |  |

**Additional file**

**Blank data collection form**

**Front Page**

**Back Page**

**Group Details**

| **FIRST NAME** | **LAST NAME** | **AREA OF FOCUS (Check all that apply)** |
| --- | --- | --- |
|  |  | I Implementing Partner Research Policy |
|  |  | I Implementing Partner Research Policy |
|  |  | I Implementing Partner Research Policy |
|  |  | I Implementing Partner Research Policy |
